# Exercise as a tool to mitigate metabolic disease

**DOI:** 10.1152/ajpcell.00144.2024

**Published:** 2024-07-09

**Authors:** Joao Victor Esteves, Kristin I. Stanford

**Affiliations:** ^1^Dorothy M. Davis Heart and Lung Research Institute, The Ohio State University Wexner Medical Center, Columbus, Ohio, United States; ^2^Division of General and Gastrointestinal Surgery, Department of Surgery, The Ohio State University Wexner Medical Center, Columbus, Ohio, United States

**Keywords:** adipose tissue, insulin resistance, liver, metabolism, skeletal muscle

## Abstract

Metabolic diseases, notably obesity and type 2 diabetes (T2D), have reached alarming proportions and constitute a significant global health challenge, emphasizing the urgent need for effective preventive and therapeutic strategies. In contrast, exercise training emerges as a potent intervention, exerting numerous positive effects on metabolic health through adaptations to the metabolic tissues. Here, we reviewed the major features of our current understanding with respect to the intricate interplay between metabolic diseases and key metabolic tissues, including adipose tissue, skeletal muscle, and liver, describing some of the main underlying mechanisms driving pathogenesis, as well as the role of exercise to combat and treat obesity and metabolic disease.

## Graphical abstract

**Figure:**
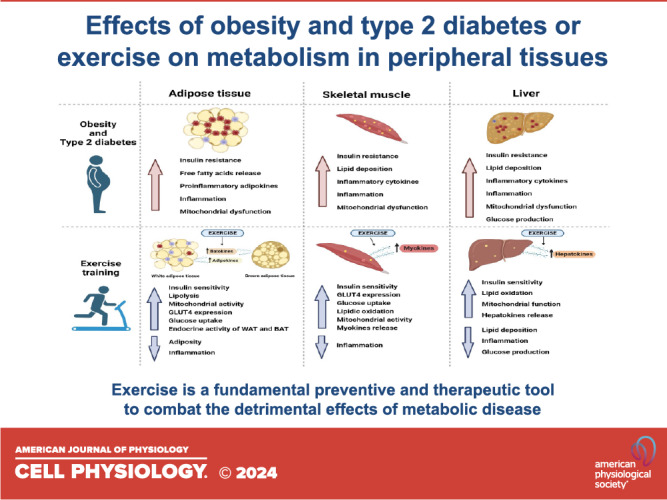

## INTRODUCTION

The growing prevalence of metabolic diseases, including obesity, type 2 diabetes (T2D), and hypertension, among others, has reached alarming proportions in recent decades, becoming a global burden (1). Specifically, regarding obesity and type 2 diabetes, it is estimated that more than 650 million adults worldwide have obesity (body mass index ≥ 30 kg/m^2^), and over 537 million adults are living with type 2 diabetes (2, 3). Substantial evidence has shown that sedentary behavior or insufficient levels of physical activity are key factors involved in the development of metabolic diseases and contribute to shortened life expectancy (4–6).

Exercise training has a central role in the prevention and treatment of chronic diseases, including obesity and T2D. There are numerous beneficial effects and adaptations of exercise for metabolic health including, but not limited to, improvements in glucose tolerance, insulin sensitivity, redox health, adaptations to the gut microbiota, and reduced inflammation (7–10). Furthermore, positive adaptations to exercise are observed in several metabolic tissues, notably in skeletal muscle, adipose tissue, and liver (11). In this review, we will discuss the relationship between obesity and type 2 diabetes, how these conditions affect metabolic tissues, and how exercise-induced adaptations in the adipose tissue, skeletal muscle, and liver improve metabolic health.

## THE RELATIONSHIP BETWEEN OBESITY AND TYPE 2 DIABETES AND THE EFFECTS OF EXERCISE

It is well established that there is a significant relationship between obesity and the development of insulin resistance in peripheral tissues and, consequently, type 2 diabetes. There are several proposed mechanisms involved in this process, including inflammation, increased levels of free fatty acids in the circulation, and mitochondrial dysfunction, all of which may play an important role.

In contrast, there are numerous adaptations in response to exercise that can combat obesity and metabolic disease. This includes improvements in blood pressure, circulating lipid profile, inflammatory profile, cardiorespiratory fitness, and cardiovascular biomarkers, among others (12–15). Specifically regarding obesity, exercise training is considered an effective strategy for weight and adiposity management, and it is associated with a reduction in body mass, adiposity, and cardiometabolic risk factors (16–18). In addition, regular physical activity has a profound impact on diabetes management and it is associated with enhanced insulin sensitivity, pancreatic β-cell function, and improved whole body glucose metabolism (13, 19–21), which directly promotes the improvement of glycemic control (7, 22).

The positive benefits observed with regular physical activity and exercise, especially those related to improvement in metabolic health aspects, occur primarily through adaptations to the adipose tissue, skeletal muscle, and liver. Therefore, in the next sections, we will describe the main mechanisms linking obesity, insulin resistance, and type 2 diabetes in peripheral tissues, and define some of the main exercise-induced adaptations to these metabolic tissues with a focus on their preventive and therapeutic role in metabolic health.

## ADIPOSE TISSUE

Adipose tissue is a highly dynamic tissue and has important functions, including energy storage in the form of triglycerides, protection against mechanical stress, the release of hormones and energetic substrates, among others (23). Adipose tissue can be broadly classified into two different types: white adipose tissue (WAT), whose primary functions are energy storage and insulation, and can be subdivided into subcutaneous WAT (scWAT) and visceral WAT (vWAT); and brown adipose tissue (BAT), which is a metabolically active tissue involved in thermogenesis by uniquely expressing uncoupling protein 1 (UCP1) (24–27).

### Adipose Tissue, Obesity, and Inflammation

Obesity is a chronic and complex disease characterized by an abnormal or excessive accumulation of adipose tissue. The increase in WAT mass, especially vWAT, is closely associated with the development of insulin resistance in metabolically hormone-responsive tissues, such as skeletal muscle, liver, and adipose tissue itself (28, 29). In addition, an excessive amount of WAT is associated with the release of free fatty acids, glycerol, several proinflammatory cytokines and chemokines, hormones, and other factors that are closely involved in the development of insulin resistance (30, 31).

The mechanisms linking obesity to insulin resistance and predisposition to T2D are numerous. One proposed mechanism is obesity-related inflammation. Adipose tissue contains multiple immune cells that together maintain the integrity and hormonal sensitivity of adipocytes (32). A class of immune cells present in adipose tissue are macrophages, which are critical contributors to inflammation and insulin resistance. In obesity, the number of adipose tissue macrophages increase and comprise up to 40% of all adipose tissue cells, which can be seen histologically by the formation of crown-like structures, consisting of macrophages surrounding dead adipocytes (33). Adipose tissue macrophages (ATMs) can be either pro- or anti-inflammatory and are typically termed as M1-like or as M2-like, respectively (34). The terms M1-like and M2-like are used to generally depict the proinflammatory state of recruited ATMs versus the anti-inflammatory state of resident ATMs (35). The M2-like ATMs secrete anti-inflammatory cytokines such as interleukin-10 (IL-10) and contribute to the maintenance of insulin sensitivity and adipose homeostasis (36, 37). Conversely, M1-like ATMs secrete proinflammatory cytokines including tumor necrosis factor-alpha (TNF-α), IL-1β, and IL-6 (36). In addition, the adipose tissue itself also secretes several proinflammatory adipokines/cytokines including TNF-α, IL-6, among many others, which leads to activation of classical inflammatory signaling such as c-Jun amino-terminal kinase (JNK) and nuclear factor-κB (NF-κB) pathways in different peripheral insulin-sensible tissues (30, 38, 39). Thus, the increase in the number of macrophages, as well as increased proinflammatory adipokines secreted, are hallmarks of the adipose tissue inflammation that accompanies obesity and is associated with the development of insulin resistance and metabolic disease (32, 33, 38).

### Adipose Tissue, Free Fatty Acids, and Insulin Resistance

A relevant mechanism linking obesity, insulin resistance, and T2D is the increased release of free fatty acids (FFA) in the circulation by adipose tissue. Under physiological conditions, insulin promotes the increase of glucose uptake, triglyceride synthesis, and repression of lipolysis, a process resulting in the hydrolysis of triglycerides into FFA and glycerol that are released into the circulation (40). However, once the adipose tissue expands, as in cases of obesity, excess lipids and toxic lipid metabolites including FFA, diacylglycerol, and ceramide accumulate in other metabolic tissues, leading to ectopic fat deposition and lipid-induced toxicity (lipotoxicity), and development of insulin resistance in muscle and liver (41, 42). It has been shown that individuals with obesity and T2D have elevated FFA levels in circulation (43, 44), and it is known that circulating FFAs cause insulin resistance in a dose-dependent manner in skeletal muscle and liver (45). The insulin resistance in these peripheral tissues caused by increased levels of FFA will contribute to the loss of glycemic homeostasis.

### Mitochondrial Dysfunction and Insulin Resistance, and Adipose Tissue

Mitochondria are highly dynamic intracellular organelles with multiple essential functions and play a critical role in energy metabolism. The key function of mitochondria is cellular respiration, which involves various processes, including activities of mitochondrial electron transport chain complexes and substrate oxidation through the tricarboxylic acid cycle, β-oxidation, ketogenesis, ATP synthesis, and reactive oxygen species (ROS) formation (46–49). An important mechanism linking obesity to diabetes is mitochondrial dysfunction, which leads to impairments in insulin sensitivity in target tissues and compromises pancreatic β-cell function (50). Mitochondrial dysfunction is a broad term that has been used to refer to numerous mitochondrial phenotypes, including decreased respiratory capacity and ATP production, reduced mitochondrial number, accumulated mitochondrial damage due to defects in mitophagy, and altered morphology resulting from changes in mitochondrial fission-fusion dynamic (51). In fact, growing evidence strongly supports the association of reduced mitochondrial function and an increase of reactive oxygen species leading to oxidative stress, particularly in insulin-responsive tissues such as skeletal muscle, white adipose tissue, and the liver (52–54). Particularly in adipose tissue, the mitochondrial number and activity determine the critical threshold at which FFA are released into circulation and exert their lipotoxic effects, promoting insulin resistance in peripheral tissues (46). Clinical and preclinical studies have also shown a reduction in white adipose tissue mitochondria content and activity in obesity and type 2 diabetes (55–57).

### Exercise-Induced Adaptations to White Adipose Tissue

Regular physical activity and exercise have important effects on adipose tissue morphology and function, including distinct changes in WAT and BAT. Regarding WAT, exercise can decrease adipocyte size and reduce lipid content in rodents, resulting in decreased adiposity (58, 59). In addition, exercise increases lipolysis and free fatty acid mobilization, which is important to provide metabolic substrate for increased energy demand during exercise, especially during low- to moderate-intensity activities and increased duration. Increased lipolysis is observed during bouts of both endurance and resistance exercise in nonobese individuals and in individuals with obesity (60, 61).

Exercise increases mitochondrial activity and increases the expression of several important metabolic proteins in white adipose tissue, including glucose transporter type 4 (GLUT4) and peroxisome proliferator-activated receptor γ coactivator 1-α (PGC1α) (58, 62, 63). Exercise training, even performed over a short period (2 wk), improves adipose tissue metabolism, including increases in glucose uptake in subcutaneous WAT and visceral WAT in both healthy and insulin-resistant individuals (64). Moreover, exercise training-induced decreases in adipocyte size and lipid content and increases in GLUT4 and PGC1α expression have been reported in both scWAT and vWAT (59, 65, 66). Fundamentally, several of these metabolic adaptations to adipose tissue can take place independently of significant weight loss showing that adipose tissue can be an important contributor to metabolic health, regardless of alterations in body weight (66). In rodents, exercise training at room temperature induces a “beiging” of scWAT, characterized by increased thermogenic and mitochondrial genes and the presence of adipocytes with multilocular lipid droplets (58, 67, 68), although this is not seen when mice are exercised at thermoneutrality. Most human studies indicate that there is no exercise-induced beiging of scWAT in humans (69–71).

Some beneficial effects of exercise can be mediated by tissue-to-tissue communication, as observed in adipose-muscle tissue cross talk (72). For instance, our laboratory has reported that transplantation of scWAT from exercise-trained donor mice into sedentary recipient mice results in improved glucose homeostasis in the recipient mice (58, 73); and, more recently, transforming growth factor-β2 (TGF-β2) has been identified as the adipokine responsible for the beneficial effects of exercise on glucose metabolism (74), demonstrating that training-induced changes in adipose tissue may have important metabolic effects on overall metabolic health.

### Effects of Exercise on Brown Adipose Tissue

BAT is a metabolically active tissue that burns lipids and carbohydrates to generate heat, and it is characterized by a high density of mitochondria, multilocular lipid droplets, and high expression of the thermogenic protein uncoupling protein 1 (UCP1) (24, 75). Several investigations have examined the effects of exercise training on BAT, with conflicting results. For example, some studies have demonstrated that exercise increases BAT activity (76–78), whereas others indicate that exercise decreases mitochondrial activity in BAT (79–81). Recently, a human randomized controlled trial investigated the effects of a 24-wk exercise intervention combining resistance and endurance training in young sedentary adults. Despite a reduction in adiposity and enhanced muscular and cardiorespiratory fitness, exercise has no effect on BAT volume activity in young sedentary adults (82). Further studies need to be performed to clarify the putative effect of exercise training on BAT activity.

Although exercise does not seem to affect BAT volume or the ability of BAT to take up glucose, recent studies have identified an important role for exercise to promote the endocrine function of BAT through the release of batokines. The term batokines refers to BAT-derived molecules, which encompass a variety of signaling molecules including peptides, metabolites, lipids, or microRNAs, and can affect the physiology of a variety of organ systems and cell types (83). A study from our research group has identified the lipokine 12,13-dihydroxy-9*Z*-octadecenoic acid (12,13-diHOME), which is secreted from BAT in response to exercise in humans and mice and increases skeletal muscle fatty acid uptake and oxidation (72) and cardiac function (84).

### Exercise, Obesity, and Adipose Tissue

**Figure 1.**
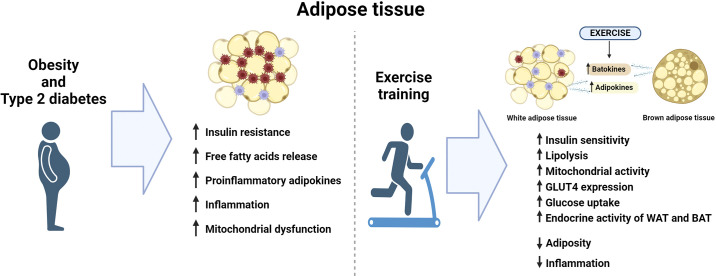
The effects of obesity and type 2 diabetes or exercise on adipose tissue. BAT, brown adipose tissue; GLUT4, glucose transporter type 4; WAT, white adipose tissue. Figure created with Biorender.com.

Although adipose tissue is directly linked to the detrimental effects of obesity on metabolic health, exercise plays a crucial role in managing these negative effects in this tissue. Exercise exerts significant effects on both white and brown adipose tissue that combat the development of obesity and metabolic disease. In WAT, the exercise-induced adaptations include decreasing adiposity and inflammation and increased lipolysis, insulin sensitivity, and enhanced metabolic activity. In BAT, exercise has an important role in promoting its endocrine function through releasing batokines that can mediate some of the positive effects of exercise (Fig. 1).

## SKELETAL MUSCLE

Skeletal muscle makes up ∼40% of the total body mass in mammals and accounts for ∼30% of the resting metabolic rate in adult humans (85). In healthy individuals, muscle accounts for around 80% of glucose disposal under insulin-stimulated conditions, as it occurs in the postprandial state (86). Skeletal muscle is considered the main tissue responsible for whole body insulin-stimulated glucose disposal and the major site of peripheral insulin resistance (87).

### Skeletal Muscle, Obesity, and Inflammation

There are several mechanisms for the development of obesity-induced insulin resistance in skeletal muscle, and inflammation has been proposed to play a role. Growing evidence indicates obesity-induced inflammation occurs in skeletal muscle through proinflammatory pathways activation with increased immune cell infiltration, particularly macrophages and T lymphocytes (88, 89). In addition, obesity is associated with increased muscle inflammatory gene expression and may alter the secretion of different cytokines/myokines (90–92). Similar to visceral fat, muscle macrophages are increased in obesity in the intermyocellular/intermuscular adipose tissue (IMAT) between the muscle fibers and in perimuscular adipose tissue (PMAT) (89). These IMAT and PMAT macrophages exhibit a proinflammatory, M1-like phenotype, and contribute to higher levels of proinflammatory cytokines, such as TNFα, IL-6, IL-1β, and C-C motif chemokine 2 (CCL2)/monocyte chemoattractant protein (MCP)-1 in skeletal muscle (93). Thus, in obesity, through the secretion of proinflammatory molecules, immune cells may induce myocyte inflammation, adversely regulate myocyte metabolism, and contribute to local and systemic insulin resistance (35, 88, 94).

### Skeletal Muscle, Mitochondrial Health, and Insulin Resistance

Mitochondria are particularly important for skeletal muscle function given the high oxidative demands imposed on this tissue by intermittent contraction (47). Mitochondrial health is essential for the proper function of skeletal muscle and metabolic health, and a decline in skeletal muscle mitochondrial content and function is associated with insulin resistance and observed in patients with obesity and type 2 diabetes (46, 95–100), leading to the hypothesis that skeletal muscle mitochondrial dysfunction might be responsible for the development of insulin resistance (101, 102). However, conflicting studies have not observed a reduction in skeletal muscle mitochondria content or function in patients with insulin resistance (103–105). Thus, whether alterations in skeletal muscle mitochondria are a cause or consequence of insulin resistance remain an important topic of discussion (106, 107), the role of mitochondria to contribute to metabolic health is essential. A more comprehensive discussion regarding skeletal muscle mitochondria and insulin resistance is reviewed elsewhere (106, 108, 109).

### Exercise-Induced Adaptations to Skeletal Muscle

Regular physical activity and exercise lead to numerous adaptations in skeletal muscle and promote many health benefits, playing a pivotal role in glycemic control and metabolic homeostasis. A well-recognized and important adaptation in skeletal muscle led by exercise is allowing the muscle to become more efficient in generating ATP (11, 110).

In addition, exercise training, especially aerobic exercise training, augments muscle mitochondrial density and function, as well as induces changes in organelle composition (85, 111, 112). For instance, it is well established that 6 wk of aerobic training can increase 50–100% muscle mitochondrial content (113), and training volume and exercise intensity are key determinants of training-induced increases in mitochondrial content and respiration (114). These skeletal muscle exercise-induced adaptations are a hallmark of exercise training and directly contribute to better substrate utilization capacity during exercise, i.e., a decrease in carbohydrate utilization and oxidation and lactate production, and an increase in fat oxidation (110) and insulin sensitivity (115, 116).

The improvement in glycemic homeostasis is a hallmark adaptation of exercise. Exercise training enhances muscle glucose uptake and increases GLUT4 translocation and expression (115, 117). The mechanisms involved in how muscle contraction/exercise increases GLUT4 translocation and expression are complex and are regulated by a combination of several factors, including 5′-AMP-activated protein kinase (AMPK), Ca^2+^/calmodulin-dependent protein kinase II (CaMKII), and RAS-related C3 botulinum toxin substrate 1 (RAC1), among others (117, 118) for GLUT4 translocation; and activation or inhibition of enhancer and repressor transcription factors upon solute carrier family 2 member 4 (*SLC2A4*) (gene that codifies GLUT4 protein) (117, 119–121). Importantly, the improvements in glucose metabolism and increases in muscle GLUT4 content after exercise training have been showed not only in healthy individuals but also in individuals with T2D (122–124).

### Exercise-Released Myokines

An important skeletal muscle adaptation is increasing the release of myokines into circulation, which could mediate some of the beneficial effects of exercise via muscle-organ cross talk with other tissues (125, 126). Myokines are molecules that are produced, expressed, and released by muscle and exert either autocrine, paracrine, or endocrine effects in target tissues (127). Exercise promotes the release of several myokines that mediate or alter the metabolic function of other tissues, including adipose tissue, liver, bone, brain, among others (128, 129). Currently, several myokines are described as involved in exercise adaptation, and some of them are proposed to facilitate the anti-inflammatory effects of exercise and, therefore, critically counteract insulin resistance and the metabolic dysfunction observed in obesity and type 2 diabetes (130). For example, the first to be discovered and one of the most studied myokine is IL-6 (131, 132). Exercise increases the muscle IL-6 expression and secretion in a muscle-contraction proportional manner, particularly when muscle glycogen content is depleted (125, 127). It has been shown that the myokine IL-6 mediates the exercise-associated anti-inflammatory effects both acutely with each bout of exercise and as a consequence of training adaptation, including reduction in visceral adipose tissue mass (128, 133). A comprehensive list of exercise-regulated myokines is reviewed elsewhere (128, 134).

### Exercise, Obesity, and Skeletal Muscle

**Figure 2.**
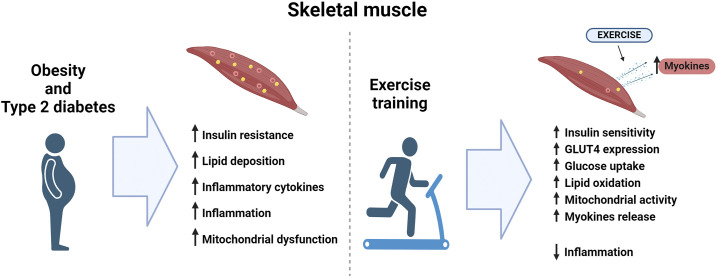
The effects of obesity and type 2 diabetes or exercise on skeletal muscle. GLUT4, glucose transporter type 4. Figure created with Biorender.com.

Exercise promotes several positive adaptations in skeletal muscle. These adaptations include increased mitochondrial activity and content, enhanced insulin sensitivity and glucose uptake, and reduced inflammation, all of which are impaired in obesity. Moreover, exercise induces the release of myokines, which act as mediators of intertissue communication and contribute to the overall metabolic benefits of exercise (Fig. 2). Thus, exercise not only enhances the function of skeletal muscle but also exerts important systemic effects, playing an essential role in combating the negative effects of obesity and type 2 diabetes on metabolic health, acting as a potent therapeutic tool.

## LIVER

The liver is one of the main metabolic organs and its dysregulation plays an important role in the development of insulin resistance and type 2 diabetes. The liver is responsible for the majority source of endogenous glucose production which, under normal postprandial rise in insulin levels, is reduced by activating hepatic glycogen synthesis and suppressing glycogenolysis and gluconeogenesis (4).

### Liver, Obesity, and Inflammation

Obesity-induced inflammation may also be observed in the liver. When the liver is insulin resistant, the inhibitory effects of insulin are impaired whereas the stimulatory effect of the hormone on lipogenesis remains intact, contributing to the development of hyperglycemia and hepatic steatosis (32, 36). Similar to adipose tissue, obesity is associated with increased hepatic inflammation and macrophages are the major source of the proinflammatory cytokines. There are two major forms of macrophages in the liver: Kupffer cells (KCs) and recruited hepatic macrophages (RHMs) (35). In obesity, the number of KCs are relatively unchanged, but there is a large increase in RHMs, which are predominantly proinflammatory (34). Although both KCs and RHMs are highly heterogeneous, RHMs express higher levels of M1-like polarized macrophage markers and proinflammatory gene expression, which is exacerbated in obesity (93). Neutrophils are another cell type that accumulates in the liver during the process of obesity and can participate in hepatic inflammation (135). Therefore, obesity is associated with increased recruitment and activation of liver macrophages, increased inflammatory signaling, and local production of inflammatory cytokines and chemokines, particularly the chemokine C-C motif chemokine 2 (CCL2), that can exert paracrine effects generating insulin resistance in hepatocytes (136, 137).

### Effects of Exercise on the Liver

Exercise has an important role to improve metabolic health and leads to several adaptations in metabolic tissues, including the liver. Considering the liver has a central role in endogenous glucose production and represents a key site involved in the development of insulin resistance and type 2 diabetes, a significant amount of literature has focused on the effects of exercise upon regulation of glycemic control and insulin sensitivity. An important exercise adaptation to the liver is to enhance the impaired insulin-induced suppressor effect upon hepatic glucose production in individuals with impaired glucose tolerance (138, 139). This is especially important to individuals with type 2 diabetes. Therefore, exercise training promotes an enhanced hepatic insulin sensitivity in individuals with obesity (140–142) and improved hepatic insulin sensitivity and suppression of hepatic glucose production in individuals with type 2 diabetes (143, 144).

As outlined earlier, obesity and type 2 diabetes are associated with increased deposition of intrahepatic lipids that can lead to nonalcoholic fatty liver disease (NAFLD) (145). In contrast, exercise training can effectively reduce intrahepatic lipids across multiple populations including individuals with obesity (146, 147), type 2 diabetes (148), and NAFLD (139, 149, 150). Also, the beneficial effects of exercise on reduction in intrahepatic lipids have been observed following different exercise interventions, such as aerobic exercise training, high-intensity intermittent exercise, combined training, among others (151, 152). It is important to mention that the reduction in intrahepatic lipids as an exercise adaptation can be realized in the absence of weight loss, although it is more powerful when significant weight loss is induced (152, 153). Exercise training also leads to increased hepatic fatty acid oxidation, improved mitochondrial function, and increases in other associated mitochondrial outcomes such as beta-hydroxyacyl-CoA dehydrogenase (β-HAD) activity, cytochrome c content, citrate synthase activity, among others in the liver (153).

### Exercise-Released Hepatokines

Another relevant role of exercise is promoting the secretion of hepatokines into blood, which can mediate metabolic adaptations to exercise training via liver cross talk with other tissues. Emerging data have identified a significant portion of hepatokines responsive to exercise intervention, but only a few have been functionally linked to the metabolic effects of exercise (10, 154). One of the hepatokines gaining increasing attention due to its potential role in mediating metabolic adaptations to exercise training is fibroblast growth factor 21 (FGF21). Several studies have demonstrated therapeutic benefits of FGF21 for obesity-related metabolic disorders, including the reduction in adiposity and improvement in insulin resistance, NAFLD, among others (155, 156). Clinical and preclinical studies have shown that circulatory levels of FGF21 are increased after acute exercise, whereas decreased after chronic exercise training (≥4 wk), due to increased FGF21 sensitivity in adipose tissue, liver, and skeletal muscle (157). It has been suggested in mice that the beneficial effects of exercise—such as the alleviation of obesity-associated insulin resistance, glucose intolerance, and ectopic lipid accumulation—are abrogated in adipocyte-specific β-klotho (FGF21 receptor) knockout (158), suggesting the important role of FGF21 in mediating the metabolic benefits of exercise (154, 158). A list including other exercise-induced hepatokines related to metabolic diseases is reviewed elsewhere (159, 160).

### Obesity, Exercise, and the Liver

**Figure 3.**
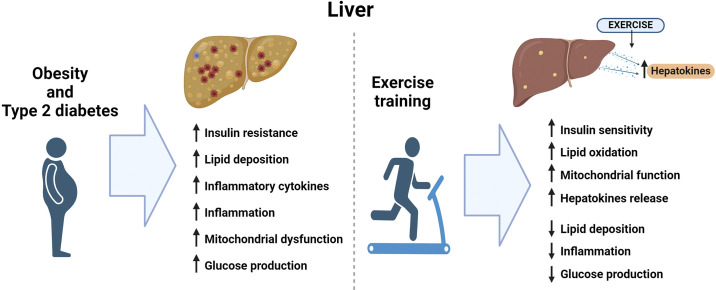
The effects of obesity and type 2 diabetes or exercise on liver. Figure created with Biorender.com.

Exercise is a potent modulator of hepatic function, leading to key adaptations that enhance metabolic health. The exercise-induced adaptations include enhancement in hepatic insulin sensitivity, effectively suppressing endogenous glucose production and reduction in intrahepatic lipid accumulation. These effects lead to improvements in hepatic lipid oxidation and mitochondrial function and counteract the general effects of obesity on hepatic function. Furthermore, exercise induces the release of hepatokines, which mediate some positive metabolic adaptations and contribute to the systemic benefits of exercise. Future research in this field is needed to elucidate the mechanisms, along with the physiological and clinical implications of exercise-released hepatokines (Fig. 3).

## EFFECTS OF EXERCISE IN PATIENTS WITH A GENETIC PREDISPOSITION TO OBESITY

In addition to environmental factors, genetics significantly contribute to the development of obesity, with heritability estimates between 40% and 70% (161). Physical exercise has also been considered a critical preventative tool in people with a genetic predisposition to metabolic disease. For instance, in patients with a genetic predisposition to obesity, exercise interventions can improve cardiorespiratory fitness and muscle strength, alter the biochemical profile (glycemia, lipid profile, and inflammatory markers), and reduce body weight (162, 163). Moreover, important populational studies have shown an inverse association between physical activity and risk for obesity, where increasing physical activity can help attenuate the genetic predisposition to obesity (164, 165). A recent study involving over 3,000 participants has found that people with an increased genetic risk of obesity require more exercise (2,300 extra steps per day) to mitigate the risk of obesity (166).

## LIMITATIONS

Exercise and metabolic disease encompass broad fields of study, and some of the significant topics had to be summarized or even omitted to be within the scope of this review. For example, the discussion on mitochondrial metabolism and dysfunction was condensed, emphasizing their roles in exercise and insulin resistance. In addition, the genetic predisposition to metabolic disease and the role of exercise were only minimally discussed. Furthermore, only a select number of signaling molecules, such as adipokines, batokines, myokines, hepatokines, and exerkines, and their role in metabolic disease/health were included in this review.

## CONCLUSIONS

In summary, several studies have shown how metabolic diseases, particularly obesity and type 2 diabetes, negatively impact metabolic health. Conversely, exercise training emerges as a pivotal preventive and therapeutic strategy with numerous positive effects, exerting profound influences across metabolic tissues. The exercise-induced beneficial adaptations in adipose tissue, skeletal muscle, and liver include enhanced lipolysis, mitochondrial activity, insulin sensitivity, glucose uptake, and reduction of intrahepatic lipids, directly contributing to the improvement of glycemic homeostasis. Furthermore, exercise-induced adaptations can be mediated by the release of molecules from metabolic tissues, termed exerkines, including adipokines from white adipose tissues, batokines from brown adipose tissue, myokines from skeletal muscle, and hepatokines from the liver. These molecules act through endocrine, paracrine, and/or autocrine pathways, facilitating tissue-to-tissue communication. Future research needs to be done to better understand the molecular mechanisms involved in the beneficial exercise-induced adaptations, and the exploration of exerkines presents a promising avenue for understanding and optimizing the therapeutic potential of exercise in metabolic health.

## GRANTS

This work was supported by NIH Grant R01DK133859-01A1 (to K.I.S.). K.I.S. and J.V.E. were also supported by the American Heart Association Grant AHA 23SFRNPCS1067042.

## DISCLOSURES

No conflicts of interest, financial or otherwise, are declared by the authors.

## AUTHOR CONTRIBUTIONS

J.V.E. and K.I.S. conceived and designed research; J.V.E. prepared figures; J.V.E. and K.I.S. drafted manuscript; J.V.E. and K.I.S. edited and revised manuscript; J.V.E. and K.I.S. approved final version of manuscript.
